# Plexiform schwannoma of the posterior tibial nerve: a case report

**DOI:** 10.4076/1757-1626-2-8392

**Published:** 2009-08-17

**Authors:** Markos Ioannou, Ioannis Papanastassiou, Ioanna Iakowidou, Stamatios Kottakis, Nikolaos Demertzis

**Affiliations:** 1 Department of Orthopaedic Surgery at Metaxa Cancer HospitalPiraeus 18537Greece; 2 Department of Pathology at Metaxa Cancer HospitalPiraeus 18537Greece

## Abstract

**Introduction:**

Plexiform schwannoma is one of the least common variants of schwannoma. It is usually found on the trunk, head, neck and upper extremities. Most reported cases are small tumors, less than 2cm in maximum diameter, arising from superficial nerves. Trauma and neurofibromatosis type 2 are well-recognized risk factors for plexiform schwannoma. It is important to differentiate it from plexiform neurofibroma, because the former has neither an association with von Recklinghausen’s disease nor a malignant potential.

**Case presentation:**

We report a case of a large plexiform schwannoma arising from the posterior tibial nerve in proximity with the medial malleolus. The patient had no history of ankle strain, fracture or neurofibromatosis type 2. Magnetic resonance imaging demonstrated a multinodular, inhomogeneous lesion, measuring 6 × 4 × 2.8 cm. Fine needle biopsy was suggestive of a benign lesion, deriving from neural elements. The mass was excised marginally. Permanent section showed that the lesion was multilobular, surrounded by a thin fibrous capsule and consisting of elongated cells, rare typical mitosis, cells with degenerative features and stained positive for S-100 protein. The patient was not evident disease at the latest follow-up 2.3 years later, with an excellent functional result. No sensory or motor deficits were encountered.

**Conclusion:**

There are no reports in the literature for large plexiform schwannomas arising from the tibial nerve. Marginal excision seems to be the recommended therapy for this rare tumor.

## Introduction

Plexiform schwannoma is a very rare, benign peripheral nerve sheath tumor, accounting for 5% of all schwannomas [1,2]. Plexiform schwannomas consist exclusively of Schwann cells presenting a plexiform arrangement which is mimicking plexiform neurofibroma, a tumor with a distinct propensity for anaplastic transformation [3]. Plexiform schwannoma was first described in 1978 by Harkin et al [4] and since then approximately one hundred cases have been reported in the literature. The tumor is most often found on the trunk, head, neck or the upper extremities, but it has occasionally been found in the lower extremities [5]. Most plexiform schwannomas reported are small and measure less than 2cm [6].

We report a case of a sizeable plexiform schwannoma of the posterior tibial nerve in proximity with the medial malleolus.

## Case presentation

**Figure 1.**
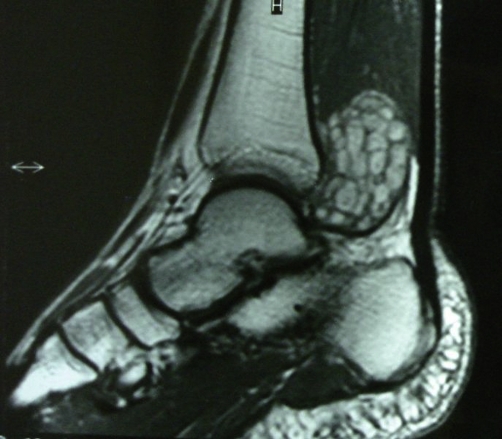
Preoperative T1-weighted MR image demonstrates a multinodular tumor of the posterior tibial nerve.

**Figure 2.**
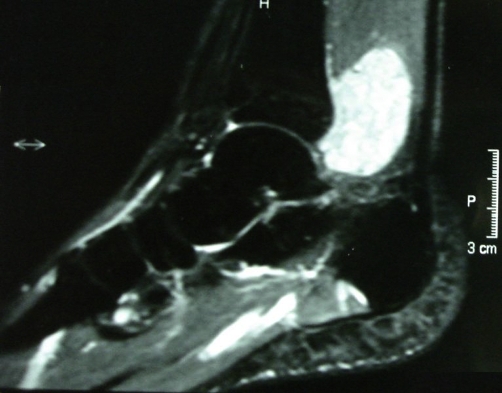
Preoperative T2-weighted MR image demonstrates a high-intensity heterogenous mass in the same place.

A 29-year-old white Caucasian male presented himself with a slowly growing mass in his right ankle. He was complaining of mild pain and paresthesia of six months duration. On physical examination a firm painless mass was palpable behind the medial malleolus. Magnetic resonance imaging (MRI) demonstrated a multinodular, inhomogeneous lesion, measuring 6 × 4 × 2.8 cm, which was isointense with muscles in T1-weighted images (Figure 1) but of very high intensity in T2-weighted images (Figure 2). No invasion of the surrounding tissues was observed. Diagnosis was oriented towards neurofibromatosis type 2 NF2. The lack of family case-history and the findings of the clinical-imaging examination (brain MRI) that ruled out the possibility of meningiomas, gliomas or schwannomas basically excluded such a diagnosis.

**Figure 3.**
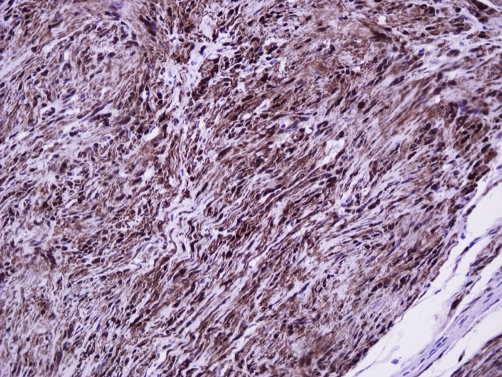
S-100 protein immunostain, magnification ×200. A consistent feature of plexiform schwannoma is a diffuse and strong positivity at S-100 protein immunostaining.

The diagnostic process was completed with fine needle biopsy which was suggestive of a benign lesion, deriving from neural elements. We performed a marginal excision of this mass and monitored the post-operative period which was uneventful. Permanent section showed that the lesion was multilobular, surrounded by a thin fibrous capsule and consisting of elongated cells, rare typical mitosis, cells with degenerative features and stained positive for S-100 protein (Figure 3) but negative for actin and desmin. The lesion was therefore considered to be a benign neurilemoma with plexiform features. The patient was not evident of disease in the latest follow-up, 2.3 years later. No sensory or motor deficits were observed and the patient was very satisfied with the outcome of the treatment.

## Discussion

Plexiform schwannoma is a rare variant of schwannoma with a plexiform or multinodular growth pattern [2,3]. This benign peripheral nerve sheath tumor appears as an asymptomatic, slowly growing lesion; it is usually found on the trunk, head, neck or upper extremities but it has exceptionally been mentioned in the lower extremities [5,7]. Most reported cases are small tumors, originating from superficial nerves. It is generally accepted that there is a strong relationship with NF2 [3,8,9] and that trauma may play a role in the formation of this tumor [3]. The main differential diagnosis includes plexiform neurofibroma as well as malignant peripheral nerve sheath tumor (MPNST) [3,10]. Differential diagnosis from plexiform neurofibroma is of paramount importance due to the fact that it has a propensity for malignant transformation, which has not been reported to occur in plexiform schwannoma [11,12]. It is also crucial to differentiate it from MPNST, which requires wide excision. In the ankle area it is frequently impossible to salvage the limb, especially if there is involvement of a major nerve/vessel, and amputation may be necessary in order to obtain wide margins [13].

## Conclusion

In summary we present a rare case of a large plexiform schwannoma of the medial malleolus arising from the main bundle of the posterior tibial nerve. The patient had no history of trauma or NF-2 which are both well-known risk factors; marginal excision yielded an excellent oncological and functional outcome.

## Abbreviations

MPNST: malignant peripheral nerve sheath tumor
MRI: magnetic resonance imaging
NF2: neurofibromatosis type 2
